# Is there a reliable size cut-off for splenic involvement in lymphoma? A [18F]FDG-PET controlled study

**DOI:** 10.1371/journal.pone.0213551

**Published:** 2019-03-08

**Authors:** Dominik Berzaczy, Alexander R. Haug, Markus Raderer, Barbara Kiesewetter, Gundula Berzaczy, Michael Weber, Marius E. Mayerhoefer

**Affiliations:** 1 Department of Biomedical Imaging and Image-guided Therapy, Division of General and Pediatric Radiology, Medical University of Vienna, Vienna, Austria; 2 Department of Biomedical Imaging and Image-guided Therapy, Division of Nuclear Medicine, Medical University of Vienna, Vienna, Austria; 3 Department of Internal Medicine I, Medical University of Vienna, Vienna, Austria; Ente Ospedaliero Cantonale, SWITZERLAND

## Abstract

**Purpose:**

Aim of present study was to determine whether the currently recommended 13-cm cranio-caudal diameter cut-off on CT for assessment of splenic involvement in lymphoma offers adequate sensitivity and specificity.

**Materials and Methods:**

Patients with histologically proven lymphoma who had undergone [18F]FDG-PET/CT before therapy were included. Cranio-caudal diameters of the spleen were measured on the CT component of PET/CT, and ROC analyses with calculation of respective areas under the curve (AUC) were used to determine cut-off values of cranio-caudal measurements with their respective sensitivities and specificities, using [18F]FDG-PET as the reference standard.

**Results:**

In 93 patients, we found a sensitivity of 74.1% and a specificity of 47% for the 13-cm splenic diameter cut-off.

**Conclusions:**

Our results show reasonable, though far from perfect sensitivities and specificities for the currently recommend 13-cm splenic diameter cut-off.

## Introduction

In lymphoma patients, diagnostic imaging using the modified Ann Arbor classification still represents the backbone of disease burden assessment. Cross-sectional imaging techniques—in particular, computed tomography (CT) and positron emission tomography (PET)–play a central role within the Ann Arbor system [1]. The majority of histological lymphoma subtypes show a high glucose metabolism, and hence, 2-F-18-fluoro-2-deoxy-D-glucose ([18F]FDG)-PET, nowadays in the form of [18F]FDG-PET/CT or, less frequently, [18F]FDG-PET/MRI, is regarded as the imaging technique of choice for staging and response assessment [2]. However, access to PET/CT, and even more so, PET/MRI, is limited in comparison to CT and MRI, as hybrid scanners are typically installed at (mostly metropolitan) tertiary care centres, often with long waiting lists for these examinations. The latter factors limit access to these hybrid (i.e. metabolic and morphologic) imaging techniques, and as a consequence, CT, due to its country-wide availability and low cost, is still a very frequently used imaging test for staging and restaging of patients with lymphoma, even in those with FDG-avid subtypes that should, according to the Lugano classification [3], undergo [18F]FDG-PET/CT.

Involvement of the spleen is common in lymphoma patients, and as the spleen is considered a secondary lymphatic organ, splenic involvement extends supradiaphragmatic nodal disease (stage II) to stage III disease [2].

While solitary masses or multiple small lesions or nodules are seen in some patients, diffuse involvement is also frequently seen, and represents a diagnostic challenge [4]. Diffuse splenic involvement on [18F]FDG-PET is diagnosed through a diffusely elevated tracer uptake, whereas on CT, the current criterion [2] is organ enlargement with a cranio-caudal diameter >13 cm. However, this cut-off value is mainly based on anatomic studies in the normal population [5], but has, to our knowledge, not been evaluated in a population of lymphoma patients.

Thus, in the present study, it was our aim to determine, in patients with FDG-avid lymphomas, whether the currently recommended cut-off value of 13 cm in terms of cranio-caudal organ diameter offers adequate sensitivity and specificity for detection of diffuse splenic involvement on CT, compared to [18F]FDG-PET.

## Materials and methods

### Patients

We performed a retrospective, electronic search in our institutional database (AKIM/DocSearch) using a systematic strategy to identify patients suitable for this evaluation. We consecutively included patients in this Institutional Review Board (local ethics committee of the Medical University of Vienna, Nr.: 1385/2016 2) approved study, that were referred to our tertiary care centre between January 2010 and June 2017 for pre-therapeutic staging of histologically proven lymphoma by [18F]FDG-PET/CT. Written informed consent was obtained from all adult patients and, in case of minor participants, from their guardians, prior to imaging.

Search terms used in different combinations included lymphoma; diffuse large B-cell lymphoma or DLBCL; Hodgkin or HL or Non-Hodgkin or NHL; follicular lymphoma or FL; mantle cell lymphoma or MCL; marginal zone lymphoma or MZL; splenomegaly; splenic involvement; splenic lesion; and [18F]FDG-PET. Liver cirrhosis, positive HIV serostatus and hematologic or hematologic-oncologic diseases likely to cause splenomegaly, lack of appropriate clinical data, as well the presence of splenic solitary masses, miliary lesions on CT or FDG avid nodules were used as exclusion criteria.

### Imaging protocol

[18F]FDG-PET/CT was performed, covering the anatomy from the superior orbital rim to the upper thigh, using a 64-row multi-detector hybrid PET/CT device (Biograph TruePoint 64; Siemens, Erlangen, Germany). For PET, this scanner offers an axial field-of-view of 216 mm, a sensitivity of 7.6 cps/kBq, and a transaxial resolution of 4–5 mm. After patients had fasted for five hours, PET was performed 45–60 min after an intravenous administration of a mean of 300 MBq of [F18]FDG, with 3-min/bed position, four iterations, and 21 subsets, a 5-mm slice thickness, and a168x168 matrix, using the point-spread function (PSF)-based reconstruction algorithm TrueX. Venous-phase CE-CT was used for attenuation correction, and was obtained after the intravenous injection of 100 ml of a tri-iodinated, non-ionic contrast medium at a rate of 2 ml/s; a tube current of 120 mA; a tube voltage of 230kV; a collimation of 24x1.2 mm; a 3 mm slice thickness with a 2-mm increment; and a 512x512 matrix.

### Image analysis

In every patient the first chronologically available hybrid imaging study was chosen for the analysis. A coronal 3D-multiplanar reformation (MPR) with a slice thickness of 3 mm and a reconstruction increment of 2 mm for the abdomen was used to determine the cranio-caudal spleen diameter (“vertical length“), as recommended by the Lugano criteria.

For evaluation of the [18F]FDG-PET component, maximum and mean standardized uptake values (SUVmax and SUVmean) were recorded using the Syngo MultiModality Workplace environment (Siemens, Erlangen, Germany). This was done by placing spherical VOIs with a 2-cm diameter in a lesion-free part of the liver and spleen parenchyma, respectively.

### Reference standard and statistical analysis

Receiver operating characteristic (ROC) analyses with calculation of respective areas under the curve (AUC) were used to determine cut-off values of cranio-caudal spleen measurements, and respective sensitivities (Se) and specificities (Sp) as well as their 95% confidence intervals (CI), were calculated. [18F]FDG-PET served as the reference standard for these calculations: a spleen with a higher [18F]FDG uptake than that of the liver parenchyma was considered to be involved, as previously recommended [6]. Demographic and pathohistological data were also collected. Pearson correlation coefficients were used to determine the relationship between the splenic diameters and SUVmax/mean values. The specified level of significance was *p*≤.05 for all tests. All statistical analyses were performed using SPSS Statistics software Version 22.0 (IBM Corp., Armonk, NY, USA).

## Results

Our systematic search strategy yielded a total of 253 patients. Of those, 159 were excluded because PET tracers other than [F18]FDG were used; an imaging modality other than PET/CT was used; or pathohistology did not meet our inclusion criteria; totalling 89 patients. In addition, 16 patients were excluded for positive HIV serostatus, portal hypertension or hepatic cirrhosis and 26 patients presented with focal splenic lesions on CT. Twenty-nine double hits, using our search criteria, were removed.

**Fig 1:**
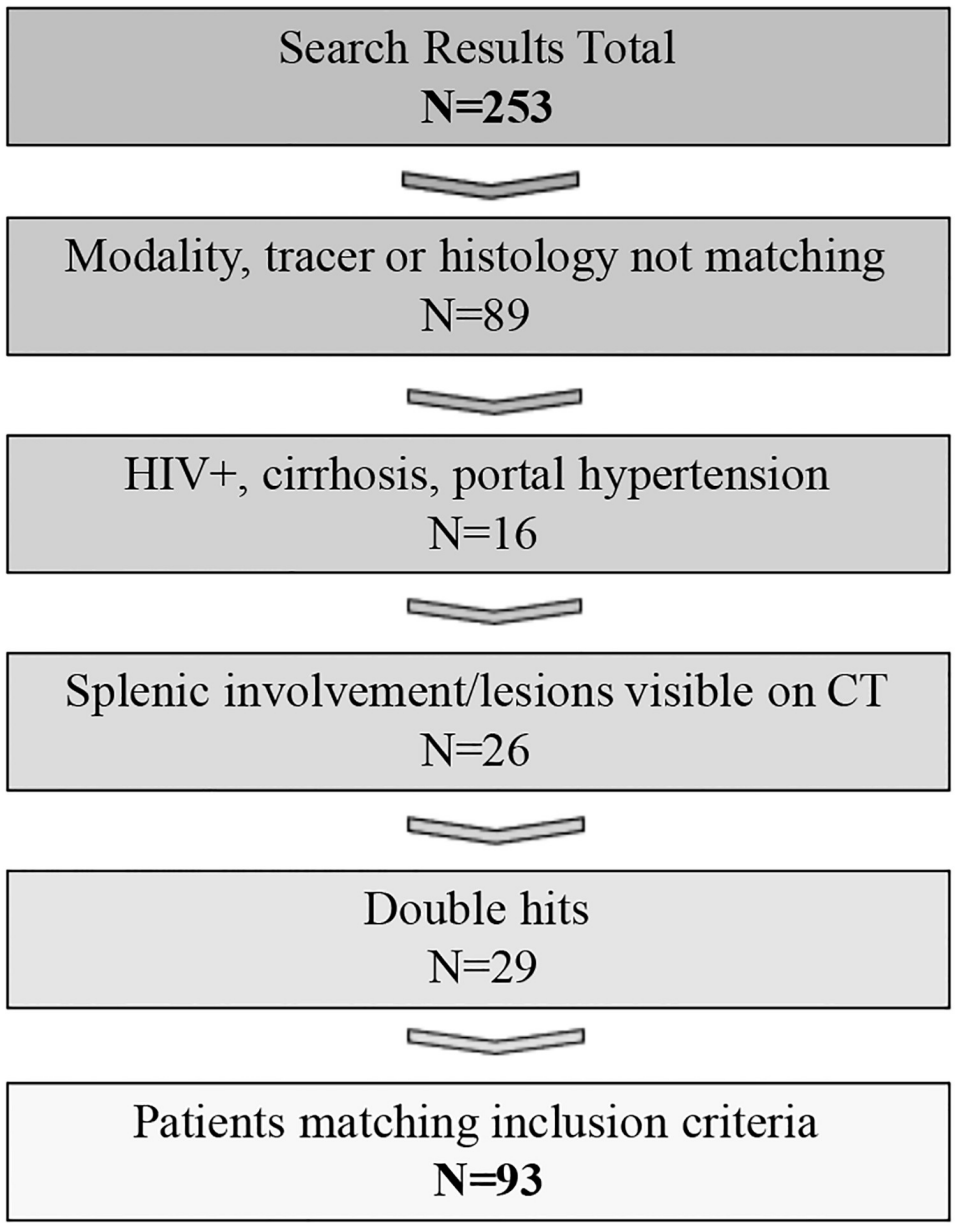
Search strategy / patient identification. Flow chart depicting results as of our inclusion und exclusion criteria.

Ultimately, 93 patients, who were referred for pre-therapeutic staging, met our criteria for participation, and were used for further analysis (see Fig 1).

Patient demographics showed a male-to-female distribution of 49 to 44, and a mean age of 48±19 years. A detailed histologic lymphoma subtype distribution among patients is shown in Table 1.

**Table 1:** Demographics and histologic subtype.

Age (mean age±SD in yrs.)	48±19	
Sex (male(n/%)/female(n/%))	(49/52.7%)/(44/47.3%)	
Histologic subtype	n	%
Hodgkin lymphoma	20	21.5
NHL (all)	32	34.4
DLBCL	13	14
Follicular lymphoma	12	12.9
Mantle cell lymphoma	7	7.5
MALT lymphoma	4	4.3
Splenic marginal zone lymphoma	3	3.2
Burkitt lymphoma	2	2.2
Total	93	100

For the currently recommended cut-off of 13 cm in cranio-caudal splenic diameter, we found a sensitivity of 74.1% (95% CI, 55.3–86.8) and a specificity of 47% (95% CI 35.4–58.8), resulting in a positive predictive value (PPV) of 36.4% (95% CI 24.9–49.6) and a negative predictive value (NPV) of 81.6% (95% CI 66.6–90.8). For lower cranio-caudal splenic diameters, sensitivity improved, but specificity declined: e.g., for a diameter of 12 cm, we found a sensitivity of 81.5% (95% CI 63.3–91.8) and a specificity of 30.3% (95% CI 20.6–42.2) resulting in a PPV of 32.4% (95% CI 22.4–44.2) and a NPV of 80% (95% CI 60.9–91.1). On the other hand, for splenic diameters of 14 and 15 cm, sensitivities were 63% and 51.9% (95% CI, 44.2–78.5 and 34–69.3), specificities were 68.2% and 81.8% (95% CI, 56.2–78.2 and 70.9–89.3), with a PPV of 44.7% and 53.8% (95% CI, 30.1–60.3 and 35.5–71.2) and a NPV of 81.8% and 80.6% (95% CI, 69.7–89.8 and 69.6–88.3), respectively.

Correlation of the splenic diameter and SUVmax and SUVmean showed a moderate degree of correlation between the groups including lymphomas with high FDG avidity (HL, DLBCL, Burkitt) and low-to-moderate FDG avidity (MCL, FL, MZL, MALT, Mycosis fungoides). In the high FDG avidity group we found *r* = .370 (*p* = 0.002) between the vertical diameter and SUVmean, and *r* = .360 (p = 0.003) between the vertical diameter and the SUVmax. The low-to-moderate FDG avidity group showed *r* = .310 (*p* = 0.12) between the vertical diameter and the SUVmean, and *r* = .430 (*p* = 0.029) between the vertical diameter and SUVmax.

**Fig 2:**
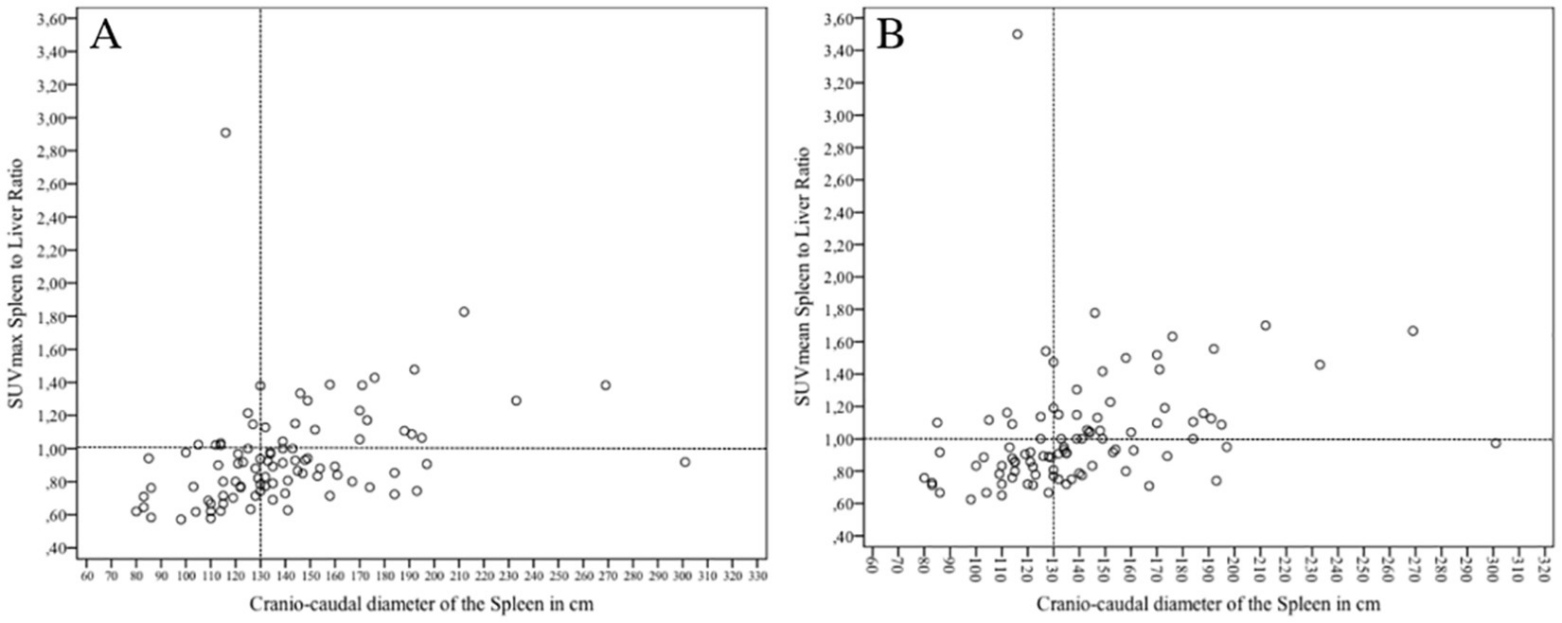
Scatter diagram of glucose metabolism vs. organ diameter. Plot depicting correlation of the SUVmax (A) and mean (B) of the spleen to liver ratio over the splenic cranio-caudal organ diameter (in cm).

Overall, we found a weak (*r* = . 320) but statistically significant (*p* = .002) correlation between the cranio-caudal diameter of the spleen and the corresponding SUVmean and SUVmax values (Fig 2).

## Discussion

Splenic involvement is frequently encountered in lymphomas, in the majority of cases together with lymph node involvement. However, isolated splenic involvement in the absence of nodal or extranodal involvement visible on CT can occur, particularly in selected histological NHL subtypes such as mantle cell lymphoma (MCL); splenic marginal zone lymphoma (SMZL) and small-cell lymphocytic lymphoma / chronic lymphocytic leukemia (SLL/CLL); hairy cell leukemia (HCL); hepatosplenic T-cell lymphoma (HSTL); lymphoplasmacytic lymphoma (LPL); T-cell large granular lymphocytic leukemia (T-LGL); and B-cell prolymphocytic leukemia (B-PLL) [7]. Notably, some of the latter subtypes, such as MZLs, SLL/CLL, and LPL, are known to show a variable FDG avidity, and as a consequence, the Lugano guidelines generally do not recommended the use of [18F]FDG-PET/CT or -PET/MRI in those subtypes, but instead, recommend CE-CT [8]. In view of the above, and because CT is often the first cross-sectional imaging test used in lymphoma patients due to the country-wide access to this technique, a reliable CT criterion for assessment of splenic involvement in lymphoma patients is desirable [9,10].

**Fig 3:**
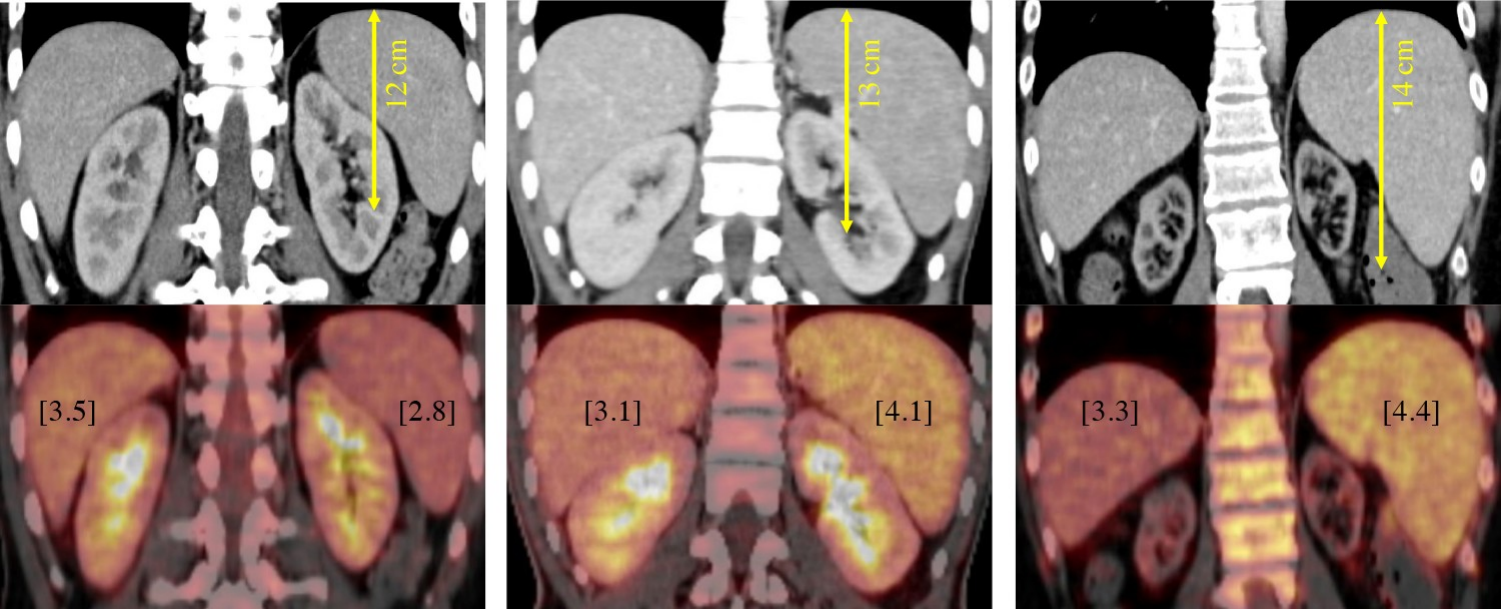
Correlation of glucose metabolism vs. organ diameter. Increasing glucose metabolism (from left to right) expressed through the SUVmax (in square brackets) shows a weak but statistical significant correlation to cranio-caudal organ diameter in three different patients.

**Fig 4:**
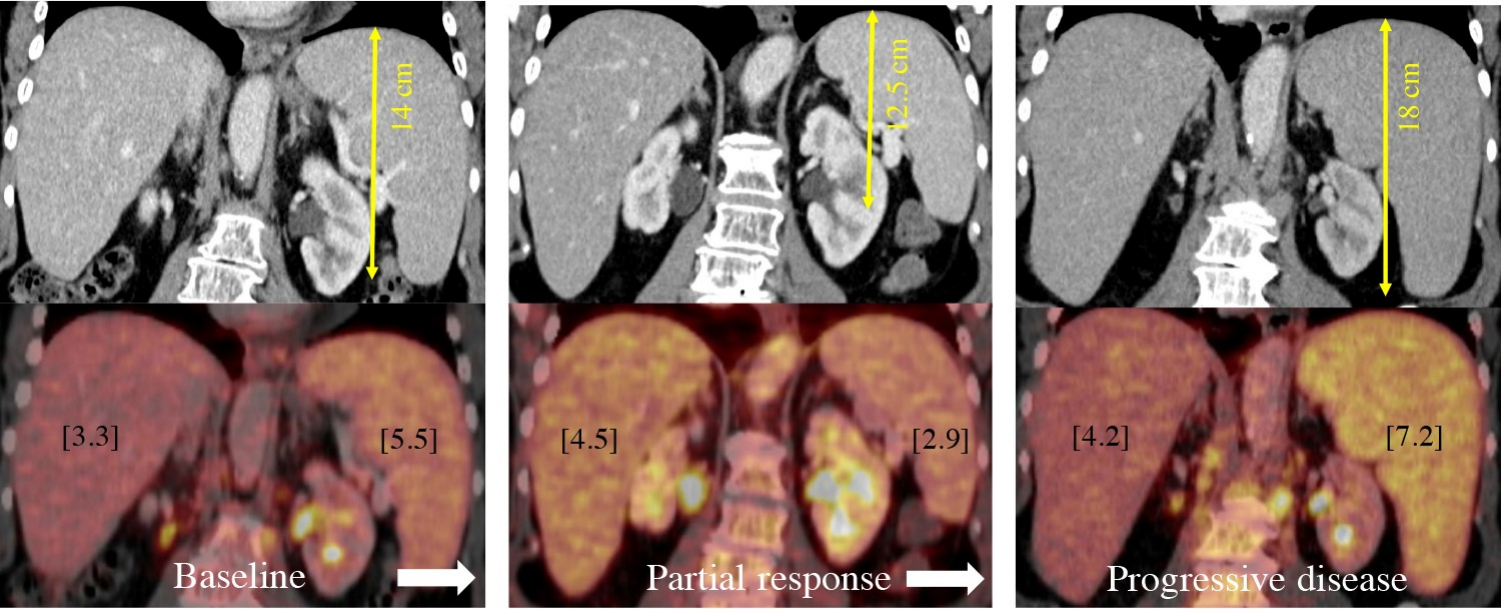
Change of organ diameter and corresponding SUV max during therapy. 44-year-old male patient suffering from DLBCL from baseline (left) and follow-up (mid and right) showing the correlation of higher glucose uptake and consecutive higher cranio-caudal splenic diameter during partial response and progressive disease over time.

Both the Lugano classification and the recently proposed RECIL (International Working Group consensus response evaluation criteria in lymphoma) criteria recommended the use of a 13-cm vertical length as a uniform cut-off for detection of splenic involvement) in lymphoma patients, regardless of the histological lymphoma subtype (Figs 3 and 4) [2,11]. For conformity with these guidelines, we also did not further subdivide our sample into histological subtypes for calculation of sensitivities and specificities.

However, we are not aware of any previous studies that formally evaluated the 13-cm cut-off, or compared it to other cut-offs, in terms of sensitivity or specificity, and which can thus serve as a basis for this recommendation. A look at our results clearly shows that, while the sensitivity of the 13-cm cut-off is adequate, the specificity of slightly less than 50% is not sufficient to rule out the presence of splenic lymphoma. For clinical practice, this means that patients with clinical suspicion of lymphoma (due to patient history and symptoms) in the absence of splenomegaly or lymphadenopathy on CT should routinely undergo bone marrow biopsy in addition to peripheral blood analysis and flow cytometry for immunophenotypic, biomolecular, and cytogenetic analysis. In case of findings that are concordant with lymphoma, [18F]FDG-PET/CT or [18F]FDG-PET/MRI should be performed.

The vertical length of the spleen showed only a weak correlation with glycolytic activity, as expressed by [18F]FDG-PET-based SUVs. One possible explanation for this finding is that different histological lymphoma subtypes were included in our sample, and it is well-known that aggressive subtypes such as DLBCL show a much higher FDG avidity than indolent subtypes, such as MCL and FL. Furthermore, another possibly influencing factor—the density of the lymphoma cell infiltrate in the spleen—was not taken into account, as such a measurement would have required biopsy or resection of the organ, which was not clinically indicated [12]. Finally, only the vertical length of the spleen was measured, as recommended in the guidelines; however, it is quite possible that in a subset of patients, the transaxial diameter would have exceeded the vertical diameter.

Our study is mainly limited by the fact that our sole reference standard was [18F]FDG-PET. However, [18F]FDG-PET is well established for staging of the majority of lymphoma subtypes [8,13–15]. Notably, only patients with FDG-avid lymphomas, defined by the presence of a least a single nodal or extranodal site with increased tracer uptake on [18F]FDG-PET/CT, were included. Nevertheless, this strategy prevented us from including larger numbers of patients frequently non-FDG-avid subtypes, and some subtypes, such as SLL/CLL, were not eligible for participation at all. Furthermore, patients with focal lesions were excluded, because we wanted to limit our analysis to the subset of patients that would pose a diagnostic dilemma, with regard to assessment of splenic involvement, in routine practice. Finally, it is important to note that our results in terms of splenic size are only applicable to pre-treatment scans of patients without infectious diseases such as HIV, as the latter might lead to non-malignant splenomegaly; likewise, our results are not applicable to follow-up scans after chemotherapy or administration of hematopoietic stimulating agents that might lead to splenomegaly or increased [18F]FDG uptake in the spleen [16].

There are two main conclusions that can be drawn from our study results: (1) the currently recommended vertical length (i.e., cranio-caudal diameter) cut-off value of 13 cm for detection of splenic involvement offers a reasonable sensitivity but cannot rule out the presence of lymphomatous organ infiltration, whereas specificity is clearly lacking; and thus, (2) detection of splenic involvement by means of organ diameter measurements is not reliable enough for clinical practice, at least in cases of diffuse involvement (i.e., when excluding cases in which focal lesions can be appreciated on CT). Thus, future studies that investigate other imaging parameters, such as splenic volumes or radiomics features, are warranted.

## Supporting information

S1 File S1 Dataset. (XLSX)
